# Supplementary material for: Membraneless water filtration using CO2
Source: Nat Commun. 2017 May 2;8:15181. doi: 10.1038/ncomms15181 (PMC5418569; doi:10.1038/ncomms15181)
Supplement: Supplementary Information — Supplementary Figures, Supplementary Tables, Supplementary Discussions and Supplementary References [file ncomms15181-s1.pdf]

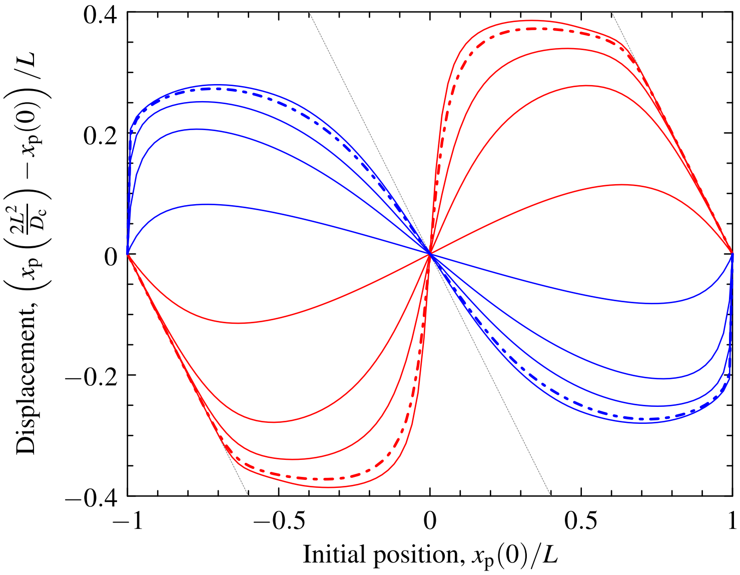

Supplementary Figure 1 | Computed displacements of (red) positively- and (blue) negatively-charged particles at several CO_2 pressures: 0.1, 1, 10, 136 (thicker dashed line), and 500 kPa in order of increasing displacement. All other parameters are as listed in Supplementary Table 1. The displacements are shown at $t = 2L^2/D_c = 168\,\text{s}$.

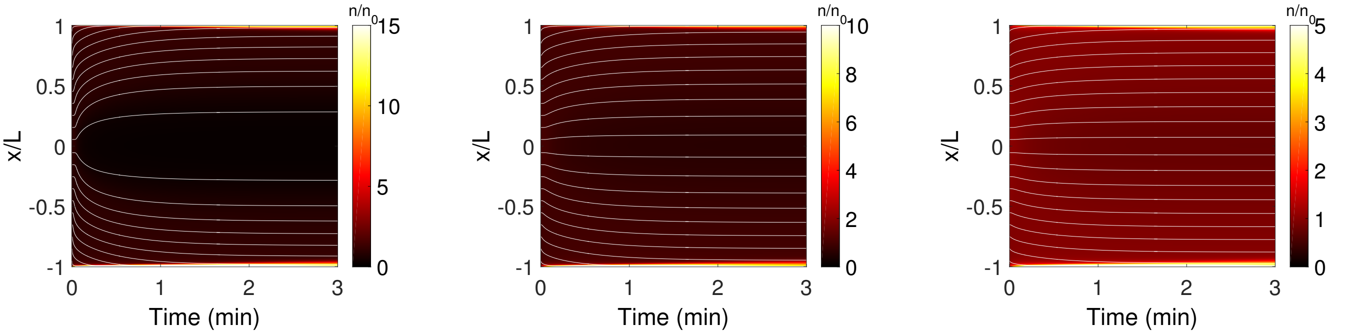

Supplementary Figure 2 | Computed particle concentrations and trajectories of positively-charged particles in (a) pure water, (b) 0.1% NaN_3 solution, and (c) 0.25% NaN_3 solution.

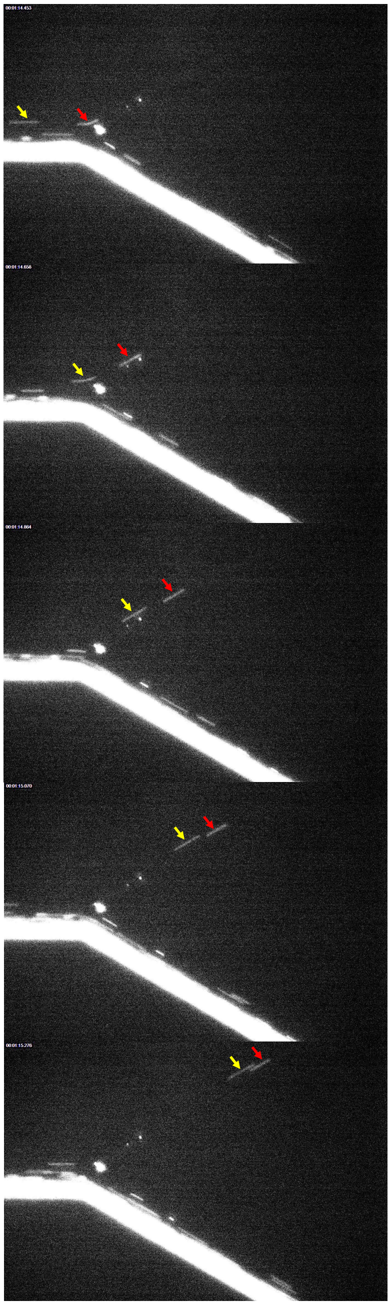

Supplementary Figure 3 | Image sequence of particle (polystyrene, 0.5 µm) removal driven by CO_2 dissolution. The time between each frame is ≈0.2 s, which allows tracking of individual particles (indicated by arrows) flowing through the filtrate stream.

Supplementary Table 1 | Parameters used in the calculations

description	variable	quantity
CO_2 diffusivity[1]	D_c	1.91×10^{-9} m^2 s^{-1}
H^+ diffusivity[1]	D_+	9.311×10^{-9} m^2 s^{-1}
$HCO_3{}^-$ diffusivity[1]	D_-	1.185×10^{-9} m^2 s^{-1}
ambipolar diffusivity	D_i	2.102×10^{-9} m^2 s^{-1}
atmospheric CO_2 partial pressure	$p^{atm}_{CO_2}$	40 Pa
applied CO_2 pressure	p_{CO_2}	135.8 kPa
channel half length	L	400 µm
particle radius	R_p	0.25 µm and 0.5 µm
particle zeta potential	ζ	−70 mV and 60 mV
CO_2 Henry's law constant[2]	K_h	2980 L kPa mol^{-1}
forward reaction rate[3]	k_f	0.039 s^{-1}
backward reaction rate[3]	k_b	9.2×10^4 L mol^{-1} s^{-1}
water viscosity	μ	0.9 mPa s

Supplementary Table 2 | Concentrations of species in water in equilibrium with CO_2 at several pressures

p_{CO_2} (kPa)	CO_2 (mol L^{-1})	H^+ (mol L^{-1})	$HCO_3{}^-$ (mol L^{-1})	$CO_3{}^{2-}$ (mol L^{-1})	OH^- (mol L^{-1})	pH
0.04	1.3×10^{-5}	2.4×10^{-6}	2.4×10^{-6}	4.7×10^{-11}	4.2×10^{-9}	5.6
1	3.4×10^{-4}	1.2×10^{-5}	1.2×10^{-5}	4.7×10^{-11}	8.4×10^{-10}	4.9
10	3.4×10^{-3}	3.8×10^{-5}	3.8×10^{-5}	4.7×10^{-11}	2.7×10^{-10}	4.4
100	3.4×10^{-2}	1.2×10^{-4}	1.2×10^{-4}	4.7×10^{-11}	8.4×10^{-11}	3.9
136	4.6×10^{-2}	1.4×10^{-4}	1.4×10^{-4}	4.7×10^{-11}	7.2×10^{-11}	3.9
500	1.7×10^{-1}	2.7×10^{-4}	2.7×10^{-4}	4.7×10^{-11}	3.7×10^{-11}	3.6
1000	3.4×10^{-1}	3.8×10^{-4}	3.8×10^{-4}	4.7×10^{-11}	2.7×10^{-11}	3.4

Supplementary Discussion

Governing equations. We use a one-dimensional model to describe the coupled diffusion and reaction of dissolved species and the diffusiophoretic motion of particles. The parameters in the model are listed in Supplementary Table 1. We neglect diffusioosmosis due to the wall surface charge since a Poiseuille flow driven by back pressure cancels the diffusioosmotic flow, making a zero net fluid flow in any given cross-section of the channel[4]. Not only is this approximation valid for the stationary experimental case in Fig. 2, but it is also true for the continuous flow filtration device in Fig. 4 because the ion gradients are established in the direction transverse to the main flow direction. Furthermore, the large speed of the main flow ($\approx 300\,\mu\mathrm{m\,s^{-1}}$) compared to diffusioosmotic flow, $O(10\,\mu\mathrm{m\,s^{-1}})$, would allow one to neglect the diffusioosmotic flow when predicting the particle motion in the filtration devices.

Using the sign convention for x presented in Figure 1, CO_2 diffuses inward from $x = \pm L$. Dissolved CO_2, concentration $c_c(x,t)$, reacts with water according to the overall reaction[5,6]

$$CO_2 + H_2O \underset{k_b}{\overset{k_f}{\rightleftharpoons}} H^+ + {HCO_3}^- \tag{1}$$

with forward rate constant k_f and backward rate constant k_b. The net forward reaction rate is $r = k_f c_c - k_b c_{H^+} c_{{HCO_3}^-}$. The transport equation for CO_2 is therefore

$$\frac{\partial c_c}{\partial t} = D_c \frac{\partial^2 c_c}{\partial x^2} - r \tag{2}$$

The transport equation for the anions and cations is

$$\frac{\partial c_\pm}{\partial t} = -\frac{\partial j_\pm}{\partial x} + r_\pm \tag{3}$$

where $j_\pm$ is the ion flux and the subscripts refer to the sign of the ion's charge. The stoichiometry of reaction 1 makes $r_+ = r_- \equiv r$. The ion fluxes are[7]

$$j_\pm = -D_\pm \left(\frac{\partial c_\pm}{\partial x} \pm c_\pm \frac{ze}{k_B T} \frac{\partial \phi}{\partial x} \right) \tag{4}$$

where ϕ is the electric potential, $z = 1$ is the ion valence, e is the elementary charge, k_B is the Boltzmann constant, and T is the absolute temperature. Under the assumption of local charge neutrality, we have $c_+ = c_- \equiv c_i$ and $j_+ = j_- \equiv j_i$. The assumption of local charge neutrality can be justified whenever the Debye length is significantly smaller than the length scale of concentration gradients[8]; at 136 kPa the Debye length is 25 nm, which satisfies this requirement. It follows that

$$j_i = -D_+ \left(\frac{\partial c_i}{\partial x} + c_i \frac{ze}{k_B T} \frac{\partial \phi}{\partial x} \right) = -D_- \left(\frac{\partial c_i}{\partial x} - c_i \frac{ze}{k_B T} \frac{\partial \phi}{\partial x} \right) \tag{5}$$

and the electric field $E = -\partial\phi/\partial x$ is

$$E = \beta \frac{k_\mathrm{B} T}{ze} \frac{\partial \ln c_\mathrm{i}}{\partial x} \tag{6}$$

where $\beta = (D_+ - D_-)/(D_+ + D_-)$. The ion flux therefore simplifies to

$$j_\mathrm{i} = -\left(\frac{2D_+ D_-}{D_+ + D_-} \right) \frac{\partial c_\mathrm{i}}{\partial x} \tag{7}$$

and we obtain

$$\frac{\partial c_\mathrm{i}}{\partial t} = D_\mathrm{i} \frac{\partial^2 c_\mathrm{i}}{\partial x^2} + r \tag{8}$$

where $D_\mathrm{i} = 2D_+ D_-/(D_+ + D_-)$ is the ambipolar diffusivity, $c_\mathrm{i}(x,t)$ is the concentration of anions or cations, and $r = k_\mathrm{f} c_\mathrm{c} - k_\mathrm{b} c_\mathrm{i}^2$.

The evolution of the particle number concentration $n(x,t)$ follows

$$\frac{\partial n}{\partial t} + \frac{\partial (u_\mathrm{dp} n)}{\partial x} = D_\mathrm{p} \frac{\partial^2 n}{\partial x^2} \tag{9}$$

where $D_\mathrm{p} = k_\mathrm{B} T/(6\pi\mu R_\mathrm{p})$ and $u_\mathrm{dp} = \Gamma_\mathrm{p} \frac{\partial \ln c_\mathrm{i}}{\partial x}$ is the diffusiophoretic speed of the particles. Accounting for the finite ratio of particle size and Debye length, we estimate the particle mobilities $-691\,\mu\mathrm{m}^2\,\mathrm{s}^{-1}$ and $1001\,\mu\mathrm{m}^2\,\mathrm{s}^{-1}$ for negatively- and positively-charged particles, respectively, based on the Keh and Wei model[9]. We do not consider variation of the mobility with the local pH since the zeta potential of polystyrene does not change significantly within the range of interest[10].

Exploiting the symmetry of the domain, we only solve for the CO_2, ion, and particle concentrations for $x \in [0, L]$. The boundary conditions for c_c, c_i, and n at $x = L$ are

$$c_\mathrm{c} = c_\mathrm{c}^\mathrm{sat}, \quad \partial_x c_\mathrm{i} = 0, \quad \partial_x n = 0 \tag{10}$$

where $c_\mathrm{c}^\mathrm{sat}$ is the CO_2 concentration in equilibrium with the applied CO_2 pressure ($p_{\mathrm{CO}_2}/K_\mathrm{h}$ where K_h is the Henry's law constant for CO_2 in water). At $x = 0$ the boundary conditions are:

$$\frac{\partial c_\mathrm{c}}{\partial x} = \frac{\partial c_\mathrm{i}}{\partial x} = \frac{\partial n}{\partial x} = 0 \tag{11}$$

The initial conditions are

$$c_\mathrm{c}(x,0) = c_\mathrm{c}^\mathrm{atm}, \quad c_\mathrm{i}(x,0) = c_\mathrm{i}^\mathrm{atm}, \quad n(x,0) = n_0 \tag{12}$$

where $c_\mathrm{c}^\mathrm{atm}$ and $c_\mathrm{i}^\mathrm{atm}$ are the concentrations of carbon dioxide and ions in equilibrium with the atmospheric CO_2 partial pressure (40 Pa), respectively, and we take $n_0 = 1$.

To compute the trajectories of particles we integrate the positions $x_\mathrm{p}(t)$ of several particles with evenly

spaced initial positions $x_\mathrm{p}(0)$ according to

$$\frac{\mathrm{d}x_\mathrm{p}}{\mathrm{d}t} = u_\mathrm{dp}(x_\mathrm{p}(t), t) \tag{13}$$

Effect of CO_2 pressure. Supplementary Figure 1 shows the computed displacements of particles under several applied CO_2 pressures. For comparison, CO_2 partial pressures in carbonated beverages range from 200 to 400 kPa[11]. Near the experimental conditions ($p_{CO_2} = 136$ kPa), the particle displacements depend weakly on the CO_2 pressure. Over the considered range of pressures, the ratios of the final and initial ion concentrations vary from 1.6 (at 0.1 kPa) to 112 (500 kPa). When the ratio is below approximately 10, which happens for 10 kPa, the displacements vary more strongly with the CO_2 pressure, though a ratio of 2 is sufficient to generate noticeable motion.

Additional species in solution. In addition to the equilibrium of dissolved CO_2 with water

$$CO_2 + H_2O \xrightleftharpoons{K_1} H^+ + HCO_3{}^- \tag{14}$$

we have the reactions

$$HCO_3{}^- \xrightleftharpoons{K_2} H^+ + CO_3{}^{2-} \tag{15}$$

and

$$H_2O \xrightleftharpoons{K_\mathrm{w}} H^+ + OH^- \tag{16}$$

where the equilibrium constants are $K_1 = k_\mathrm{f}/k_\mathrm{b} = 4.24 \times 10^{-7}\,\mathrm{mol\,L^{-1}}$, $K_2 = 4.7 \times 10^{-11}\,\mathrm{mol\,L^{-1}}$ and $K_\mathrm{w} = 10^{-14}\,\mathrm{mol^2\,L^{-2}}$. Using these equilibria, Henry's law for the relationship between the CO_2 partial pressure and the dissolved CO_2 concentration, and the constraint of electroneutrality, we can determine the concentrations of the species in solution as a function of the CO_2 pressure. The equilibrium concentrations of the dissolved species for several representative CO_2 pressures are given in Supplementary Table 2. At these conditions, the concentrations of H^+ and $HCO_3{}^-$ are effectively equal, and the concentrations of $CO_3{}^{2-}$ and OH^- are negligible. The equilibrium concentrations may therefore be estimated as

$$c^\mathrm{eq}_{H^+} = c^\mathrm{eq}_{HCO_3{}^-} = c^\mathrm{eq}_\mathrm{i} = \sqrt{p_{CO_2} K_1 / K_\mathrm{h}} \tag{17}$$

The particle suspensions employed contain several solutes. The suspension of negatively-charged particles (Bangs Laboratories) contains 2 mM sodium azide (NaN_3) as an antibiotic, and 0.1% surfactant (Tween). Upon dilution by a factor of 100 to reach a $\sim 0.01\%$ solids volume, the concentrations become 20 µM and 8 µM, respectively. These solute concentrations are neglected because Tween is nonionic and the NaN_3 concentration is 7 times smaller than the H^+ concentration in a solution saturated with CO_2 at the pressure employed. The details of the solution composition for the amine-modified, positively charged polystyrene particles are unavailable from the supplier (Sigma-Aldrich). The provided typical compositions for this product are 0.1–

0.5% surfactant and 0.2% inorganic salt. The safety data sheet for product number L9654 indicates a NaN_3 concentration of 0.1–0.25% (which is assumed to be the main inorganic salt), which corresponds to 15 mM to 38 mM. Dilution by 200× (to ∼ 0.01% solids volume) yields 77 µM to 192 µM, which is 0.55 to 1.4 times the H^+ concentration at saturation. We estimate the effect of the additional ions on particle motion by writing

$$u_{\mathrm{dp}} = \Gamma_{\mathrm{p}} \frac{\partial \ln(c_{\mathrm{s}} + c_{\mathrm{i}})}{\partial x} \tag{18}$$

where c_s is the concentration of additional ions, which reduces the speed of the particles. This reduction is partially offset by a decrease in the Debye length and therefore an increase in the mobilities of the particles, which we estimate as $1066\,\mu m^2\,s^{-1}$ to $1107\,\mu m^2\,s^{-1}$ over the range of NaN_3 concentrations. The particle trajectories computed for a solution without additional ions, with 0.1% NaN_3, and 0.25% NaN_3 are compared in Supplementary Figure 2. Due to reasonable agreement with the experimental results, the trajectories for 0.1% NaN_3 were presented in Figure 3d.

Supplementary References

1. Haynes, W. M. (ed.) *CRC Handbook of Chemistry and Physics* (CRC Press, 2015), 96th edn.
2. Yaws, C. L. *Handbook of Properties for Environmental and Green Engineering* (Gulf Publishing Company, 2008), 1st edn.
3. Jolly, W. L. *Modern Inorganic Chemistry* (McGraw-Hill, New York, 1991), 2nd edn.
4. Shin, S. *et al.* Size-dependent control of colloid transport via solute gradients in dead-end channels. *Proc. Natl. Acad. Sci. U.S.A.* **113**, 257–261 (2016).
5. Ho, C. & Sturtevant, J. M. The kinetics of the hydration of carbon dioxide at 25°. *J. Biol. Chem.* **238**, 3499–3501 (1963).
6. Gibbons, B. H. & Edsall, J. T. Rate of hydration of carbon dioxide and dehydration of carbonic acid at 25°. *J. Biol. Chem.* **238**, 3502–3507 (1963).
7. Cussler, E. L. *Diffusion: Mass Transfer in Fluid Systems* (Cambridge University Press, Cambridge, UK, 2009), 3rd edn.
8. Jackson, J. L. Charge neutrality in electrolytic solutions and the liquid junction potential. *J. Phys. Chem.* **78**, 2060–2064 (1974).
9. Keh, H. J. & Wei, Y. K. Diffusiophoretic mobility of spherical particles at low potential and arbitrary double-layer thickness. *Langmuir* **16**, 5289–5294 (2000).
10. Ohshima, H. & Furusawa, K. *Electrical Phenomena at Interfaces: Fundamentals, Measurements, and Applications* (Dekker, New York, 1998), 2nd edn.
11. Kuntzleman, T. S. & Richards, C. Another method for determining the pressure inside an intact carbonated beverage can (or bottle). *J. Chem. Educ.* **87**, 993 (2010).
